# Location-Dependent Excitatory Synaptic Interactions in Pyramidal Neuron Dendrites

**DOI:** 10.1371/journal.pcbi.1002599

**Published:** 2012-07-19

**Authors:** Bardia F. Behabadi, Alon Polsky, Monika Jadi, Jackie Schiller, Bartlett W. Mel

**Affiliations:** 1 Department of Biomedical Engineering, University of Southern California, Los Angeles, California, United States of America; 2 Synaptic Physiology Section, National Institute of Neurological Disorders and Stroke, National Institutes of Health, Bethesda, Maryland, United States of America; 3 Computational Neurobiology Laboratory, Salk Institute for Biological Studies, La Jolla, California, United States of America; 4 Department of Physiology, Technion Medical School, Bat-Galim, Haifa, Israel; 5 Neuroscience Graduate Program, University of Southern California, Los Angeles, California, United States of America; École Normale Supérieure, College de France, CNRS, France

## Abstract

Neocortical pyramidal neurons (PNs) receive thousands of excitatory synaptic contacts on their basal dendrites. Some act as classical driver inputs while others are thought to modulate PN responses based on sensory or behavioral context, but the biophysical mechanisms that mediate classical-contextual interactions in these dendrites remain poorly understood. We hypothesized that if two excitatory pathways bias their synaptic projections towards proximal vs. distal ends of the basal branches, the very different local spike thresholds and attenuation factors for inputs near and far from the soma might provide the basis for a classical-contextual functional asymmetry. Supporting this possibility, we found both in compartmental models and electrophysiological recordings in brain slices that the responses of basal dendrites to spatially separated inputs are indeed strongly asymmetric. Distal excitation lowers the local spike threshold for more proximal inputs, while having little effect on peak responses at the soma. In contrast, proximal excitation lowers the threshold, but also substantially increases the gain of distally-driven responses. Our findings support the view that PN basal dendrites possess significant analog computing capabilities, and suggest that the diverse forms of nonlinear response modulation seen in the neocortex, including uni-modal, cross-modal, and attentional effects, could depend in part on pathway-specific biases in the spatial distribution of excitatory synaptic contacts onto PN basal dendritic arbors.

## Author Summary

Pyramidal neurons (PNs) are the principal neurons of the cerebral cortex and therefore lie at the heart of the brain's higher sensory, motor, affective, memory, and executive functions. But how do they work? In particular, how do they manage interactions between the classical “driver” inputs that give rise to their basic response properties, and “contextual” inputs that nonlinearly modulate those responses? It is known that PNs are contacted by thousands of excitatory synapses scattered about their dendrites, but despite decades of research, the “rules” that govern how inputs at different locations in the dendritic tree combine to influence the cell's firing rate remain poorly understood. We show here that two excitatory inputs contacting the same dendrite interact in an asymmetric nonlinear way that depends on their absolute and relative locations, where the resulting spectrum of location-dependent synaptic interactions constitutes a previously unknown form of spatial analog computation. In addition to suggesting a possible substrate for classical-contextual interactions in PN dendrites, our results imply that the computing functions of cortical circuits can only be fully understood when the detailed map of synaptic connectivity – the cortical connectome – is known down to the subdendritic level.

## Introduction

**Figure 1:**
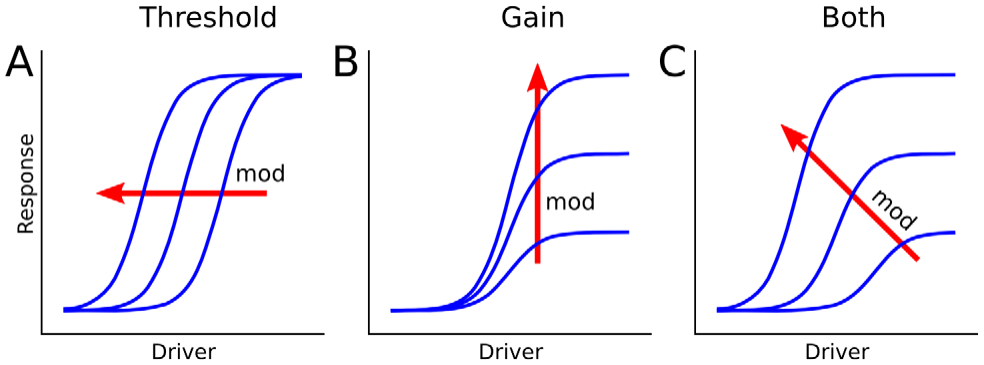
A spectrum of possible excitatory driver-modulator (classical-contextual) interactions. Conceptual curve families illustrate: ***A,*** pure threshold-lowering, ***B,*** pure gain-boosting, and ***C,*** mixed modulatory effects.

Pyramidal neurons, the principal cells of the neocortex, receive at least two broad classes of excitatory inputs. Classical driver inputs, which give rise to the neuron's basic receptive field properties, are generally associated with vertical connections from other cortical layers [1]–[3]. Non-classical excitatory inputs modulate neural responses based on sensory [4], [5], attentional [6], [7], cross-modal [8], and other “contextual” information [9], [10], and are thought to be carried by the dense network of horizontal connections within a cortical area, and feedback connections from other areas [3], [5], [11]–[13]. Conceptually, excitatory forms of modulation include pure threshold-lowering effects which left-shift a neuronal (or dendritic) input-output curve without changing its gain (Figure 1A), pure gain-boosting effects that multiplicatively scale input-output curves without changing their thresholds (Figure 1B), as well as a spectrum of mixed effects that include both threshold and gain changes (Figure 1C) [for review see 14].

Previous studies have identified a variety of mechanisms that could allow one excitatory pathway to boost a cell's responsiveness to another. Some have involved direct modulation of the soma [15]–[17], while others have focused on signal interactions through the main apical trunk, such as the coupling of apical and somatic spike-generating mechanisms [18]–[20] or the gating of distally evoked responses through the apical trunk to the soma [21]–[23]. In contrast to these relatively long range interactions that affect the entire apical tree or the cell as a whole, other studies have focused on excitatory interactions operating on a more local scale – within individual thin dendrites [24]–[33]. Among these earlier studies, however, a mechanism with the flexibility to produce a broad spectrum of excitatory classical-contextual interactions has not so far been identified.

In this work we have focused on neocortical PN basal dendrites as a possible site for classical-contextual interactions, since they receive a large fraction of a PN's excitatory input that includes both vertical and horizontal connections [2], [3], [34]. Unlike the clear distinctions between driver and modulator synapses in the thalamus [35], however, little is known regarding what features of excitatory synapses on PN basal dendrites lead their post-synaptic effects to be classical or contextual, or more fundamentally, what allows the activity level in one excitatory pathway projecting to these branches to alter the threshold or the gain, or both, of another pathway's evoked response. We hypothesized that the location-dependent cable properties of thin perisomatic dendrites [36]–[39], in concert with their intrinsic voltage-dependent membrane mechanisms [38], [40]–[42], could lead synapses near and far from the soma to modulate each other's responses in asymmetric nonlinear ways, and thus provide a possible substrate for classical-contextual interactions directly within the PN basal dendritic tree.

## Results

### Assessing the location dependence of the NMDA/AMPA peak conductance ratio

**Figure 2:**
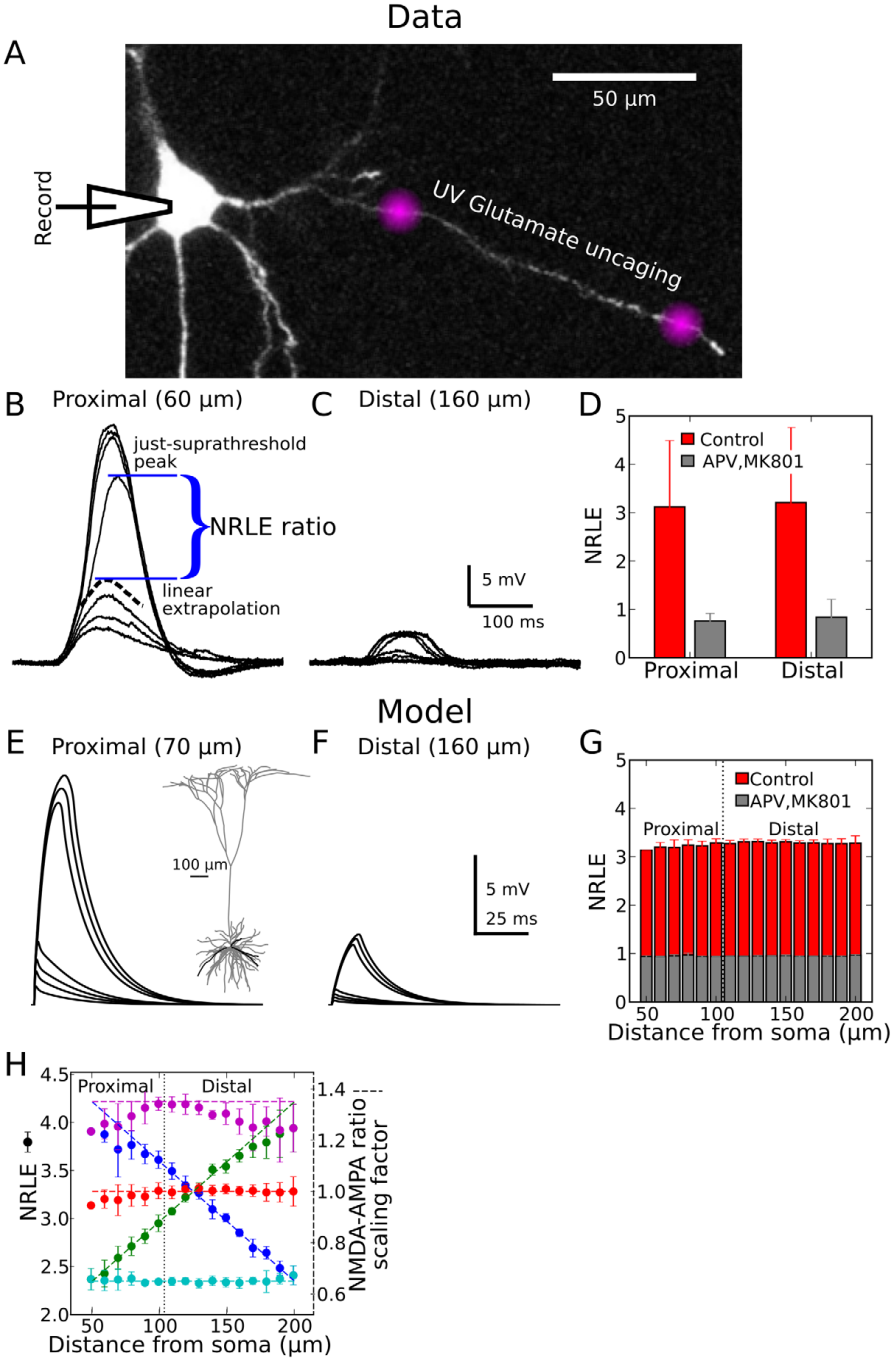
Location independence of the dendritic spike-threshold nonlinearity. ***A,*** Experimental setup. Whole-cell recordings were performed from the soma of a layer 5 pyramidal neuron. The cell was loaded with OGB-1 (200 µM) and was visualized using fluorescence confocal microscopy. Purple “clouds” denote sites of glutamate uncaging. ***B,*** Somatic responses to increasing stimulus intensity using UV laser focal uncaging of glutamate 60 µm from the soma. Black dashed trace shows extrapolated response based on linear fit to series of subthreshold response peaks. Ratio of actual to extrapolated response at local spike threshold defines the “Nonlinearity Relative to Linear Extrapolation” (NRLE) ratio (see Materials and Methods). ***C,*** Same as (***B***), but for stimulus site 160 µm from soma. ***D,*** NRLE values at proximal and distal sites were equivalent (∼3) under control conditions, and were reduced to equivalent values (<1) by NMDA channel blockers APV and MK-801. Bars indicate mean ±SD. ***E,*** Model responses at soma to increasing stimulus intensity (# of synapses) at 70 µm. ***F,*** Same as (***E***) but for stimulus at 160 µm. ***G,*** As in the experimental data, model NRLE values under control and NMDA block conditions were nearly constant along the proximal-distal axis. Error bars indicate SD across four different dendritic branches in the model, highlighted in the inset in (***E***). ***H,*** Red data are same as in (***G***). When the NMDA-AMPA ratio is made uniformly higher or lower over the length of the dendrite (magenta and cyan dashed lines, respectively), the NRLE measure roughly follows suit (magenta and cyan points). Similarly, if the NMDA-AMPA ratio increases or decreases linearly along the length of the dendrite (diagonal green and blue dashed lines, respectively), the NRLE ratio also varies roughly linearly (green and blue points).

Excitatory inputs to pyramidal neuron basal dendrites can trigger local spikes mediated primarily by *N*-methyl-d-aspartate receptor (NMDAR) channels [30], [39], [40], [43]–[45]. The location-dependence of NMDA spike properties evoked by stimulation at different distances from the soma was recently demonstrated using UV laser uncaging of glutamate onto basal dendrites of layer 5 pyramidal neurons in acute slices [44], and was further quantified herein order to set the location-dependence (or lack thereof) of the NMDA-AMPA peak conductance ratio in our compartmental model (Figure 2). Though more proximal sites generate larger somatic responses and have higher spike thresholds as expected from passive cable theory [36], we found no significant difference in a measure of the local spike-thresholding *nonlinearity* as a function of input location (Figure 2A–D). Specifically, the “nonlinearity relative to the linear extrapolation” (NRLE) was quantified at each stimulated site by finding the point along that site's input-output curve that maximized the ratio of the actual to the predicted voltage response based on a linear fit to all preceding data points (Figure 2B, see Materials and Methods for further details). Intuitively, the maximum NRLE value occurred at the largest/sharpest upturn in the input-output curve. A comparison of NRLE values is shown for proximal and distal sites in Figure 2D (red columns), with the proximal-distal cutoff at 100 µm. The difference was not significant (proximal NRLE = 3.12±1.37, N = 15 cells, 35 locations, distal NRLE = 3.21±1.55, N = 10 cells, 18 locations; p = 0.84). When NMDARs, but not AMPARs (Alpha-amino-3-hydroxy-5-methyl-4-isoxazole propionic acid) were blocked with 50 µM APV (2-amino-5-phosphonovaleric acid) and 100 µM MK-801 ((+)-5-methyl-10,11-dihydro-5*H*-dibenzo[*a*,*d*]cyclohepten-5,10-imine maleate), a complete collapse of the dendritic spike nonlinearity resulted, and NRLE scores dropped to values below 1 that were equivalent for both proximal and distal sites (proximal NRLE = 0.76±0.16, N = 10; distal NRLE = 0.84±0.37, N = 5; p = 0.71).We tuned our compartmental model to produce similarly uniform NRLE values (Figure 2E–G), which we found could be achieved by setting a spatially uniform NMDA-AMPA peak conductance ratio along the length of the branch (ratio was 2.38∶1, corresponding to the red dashed line in Figure 2H; red points show NRLE values at different distances from the soma, corresponding to the red bars in Figure 2G). In general, the choice of NMDA-AMPA ratio had a straightforward effect on NRLE scores, as shown in Figure 2H for cases with higher (magenta) or lower (cyan) uniform ratios, and cases with linearly increasing (green) or decreasing (blue) ratios. The use of a uniform NMDA/AMPA ratio in our simulations meant that any location-dependent effects produced by the model arose from synaptic interactions and nonuniform cable properties rather than differences in the synapses themselves.

### Mapping out two-input summation in the compartmental model

**Figure 3:**
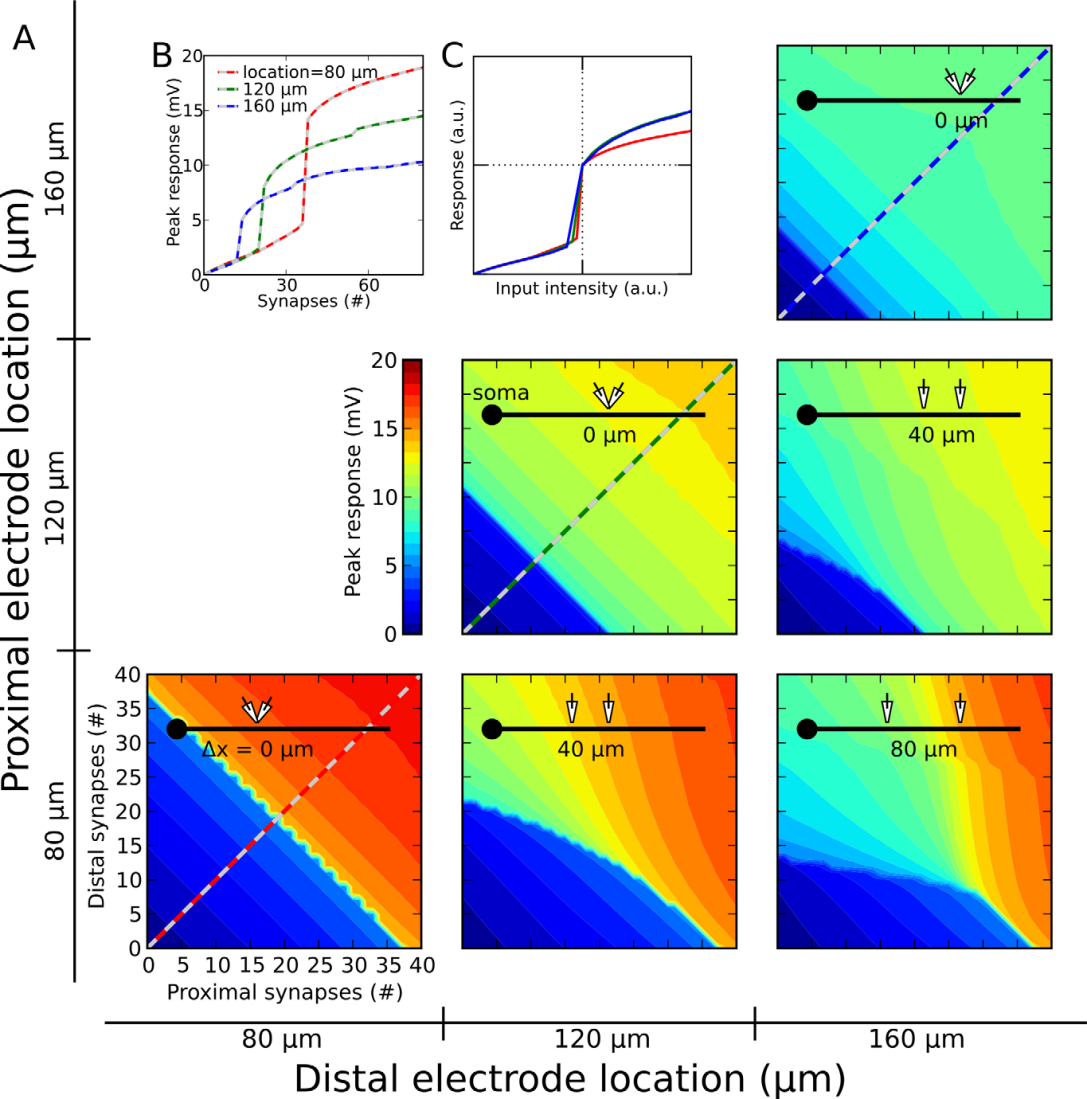
Proximal-distal interactions: predictions of the detailed compartmental model. ***A,*** Location pairs are indexed on outer x and y axes, and depicted by electrode icons in insets (number shown under electrodes is separation distance). Proximal and distal stimulus intensity is indexed on inner x and y axes, respectively, in each subplot. Striped lines in 3 subplots on main diagonal are shown in (***B***). ***B,*** Dendritic spike threshold and amplitude recorded at the soma increased markedly as electrodes approached soma. ***C,*** Superposition of curves normalized to first suprathreshold point shows nearly invariant basic shape of input-output curve. The normalization was a x,y-scaling of each curve such that the first suprathreshold data point was placed at the middle of the plot.

Using the uniform NMDA-AMPA ratio that resulted from the above fitting procedure, we used the compartmental model to map out the summation “arithmetic” for two inputs delivered simultaneously to a basal dendrite. Figure 3A shows somatic voltage responses to varying combinations of proximal and distal inputs (at 80 µm, 120 µm, and 160 µm from the soma), over a range of stimulus intensities (0 to 40 activated synapses). For convenience, numbers below each pair of schematic electrodes indicate spatial separations between the stimulus sites.

When the two stimuli were co-localized (3 subplots on main diagonal), the branch input-output function could be described by a sigmoidal (s-shaped) nonlinearity of a stereotyped form (Figure 3B). The finding of a sigmoidal input-output function was consistent with previous descriptions of synaptic integration in pyramidal neuron thin dendrites [28], [31], [39], [40], [43]–[45], as was the horizontal and vertical scaling of the input-output function depending on distance (Figure 3C) [see also 44].

**Figure 4:**
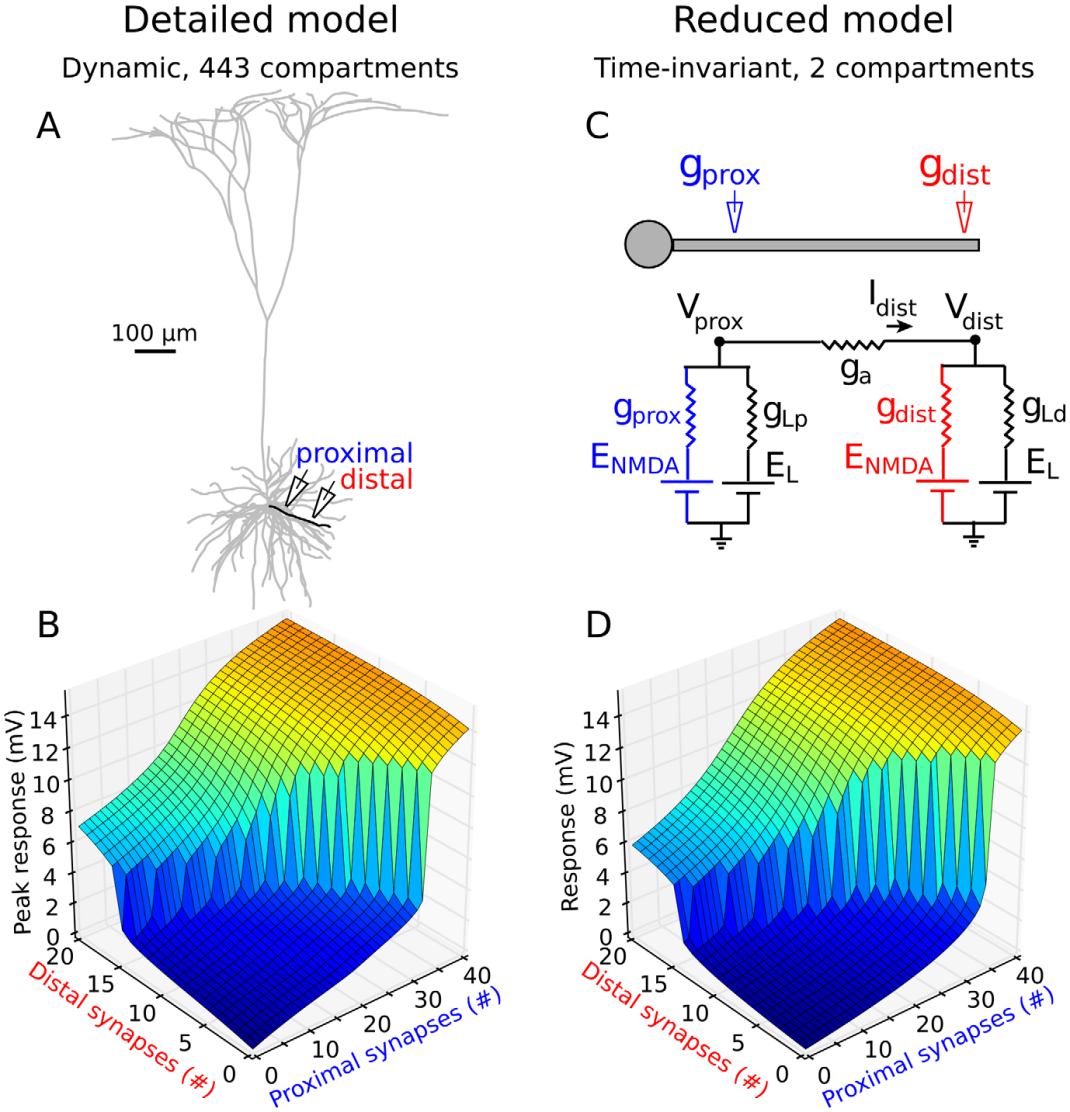
Proximal-distal interactions in a time-invariant 2-compartmental model are nearly indistinguishable from those produced by the detailed compartmental model. ***A,*** Schematic proximal and distal ‘stimulating electrodes’ are shown activating one highlighted terminal basal dendrite. ***B,*** Peak somatic responses for inputs at 90 and 150 µm as illustrated in (***A***). Plot shares color bar with Figure 3A. ***C,*** 2-compartment circuit diagram with proximal and distal NMDA conductances. ***D,*** Time-invariant responses for 2-compartment model. Parameters were hand tuned to resemble (***C***): Axial, distal leak and proximal leak conductances were 2.5,0.25, and 4 A.U., respectively. NMDA peak conductance was 0.5 A.U. per synapse. Overall peak response in 2-compartment model was scaled to match overall peak in (***C***) (V_soma_ = 15.2 mV). For details on 2-compartment model see Figure S1 in Text S1.

The pattern of responses grew markedly more complex when the two inputs were spatially separated (3 off-diagonal plots in Figure 3A). In the lower right panel of Figure 3A,for example, a 2-D sigmoidal structure was still apparent, but the proximal sigmoid (corresponding to voltage values running along the x-axis and red curves in Figure 3B, C), and the distal sigmoid (voltage responses along the y-axis and blue curves in Figure 3B, C), were now very different. This difference gave rise to an asymmetric 2-D sigmoidal function with curved, irregular contours. A representative case for stimuli at 90 and 150 µm (Figure 4A) is shown in 3-D format in Figure 4B.To determine whether this asymmetric pattern of 2-input summation depended on the detailed time courses of the AMPA and NMDA conductances, voltage-dependent Na^+^ and K^+^ currents, capacitive effects, or any other temporal dynamics in the full compartmental model, we tested whether the effects could be reproduced by a 2-compartment model containing only 5 time-invariant conductances, thus lacking any temporal dynamics at all (Figure 4C). The responses of the 2-compartment model were nearly indistinguishable from those produced by the full compartmental model (compare Figures 4B and 4D), suggesting that the proximal-distal interactions we observed depend on the voltage-dependence of the NMDA channels, and the asymmetric placement of the two stimulus sites relative to the low input-resistance soma, but not on detailed aspects of synaptic timing or other membrane dynamics (see Figure S1 in Text S1 for details and analysis).

### Experimental test of proximal-distal summation asymmetry

**Figure 5:**
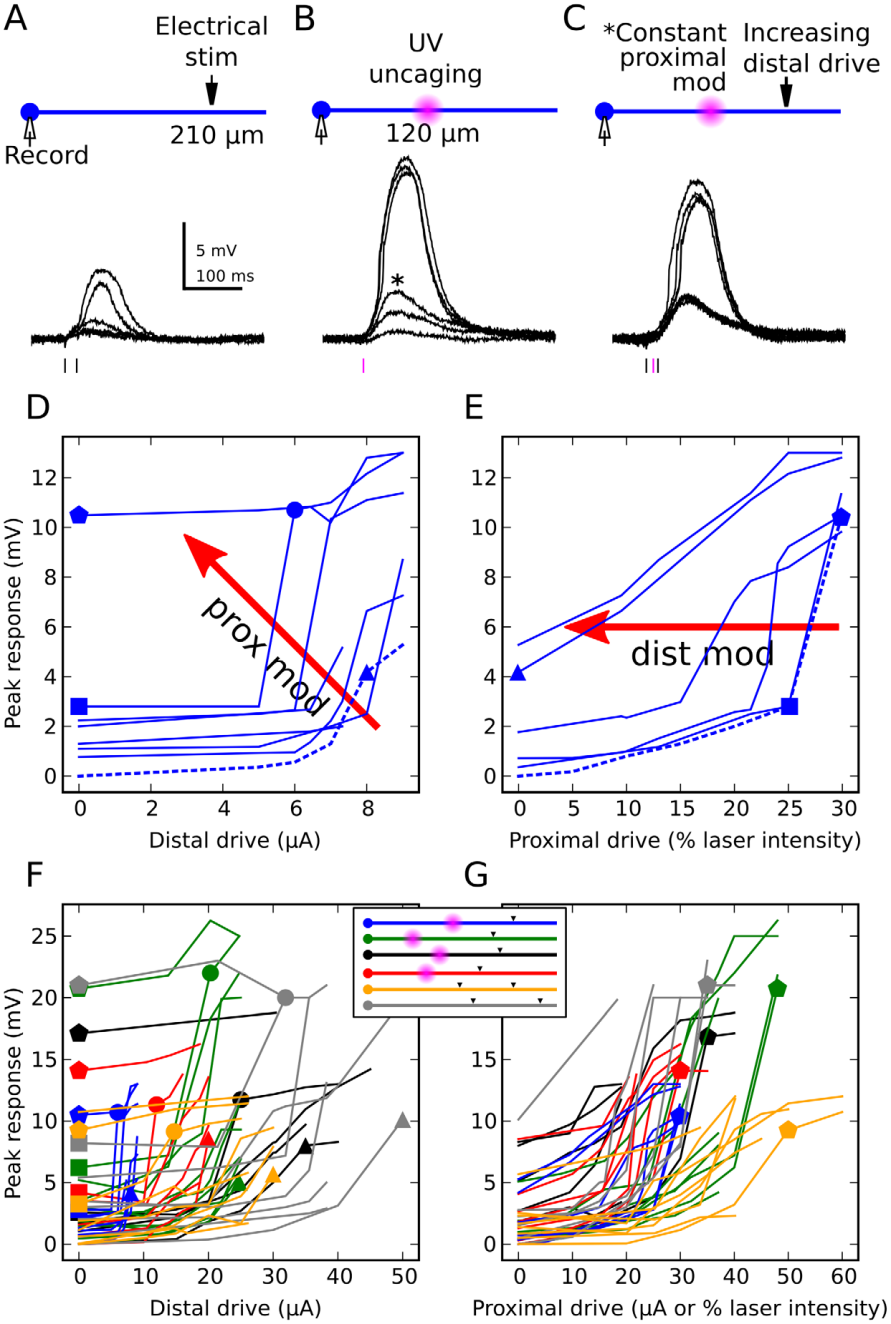
Proximal-distal interactions: experimental results. ***A,*** Somatic responses evoked by 50 Hz double pulse stimulation with bipolar theta electrode at distal site (210 µm from the soma), including a clearly visible dendritic spike. ***B,*** Somatic responses to proximal input alone at 120 µm, evoked by laser flash photolysis of caged glutamate. ***C,*** Distally evoked responses in the presence of constant proximal modulation activated simultaneously; modulatory input alone is indicated by asterisked trace in (***B***). ***D,*** Summary plot: each successive curve corresponds to a higher proximal modulation. Modulator-alone peaks are given by y-intercepts. Triangle indicates just-suprathreshold response to distal input, also shown in (***E***). Circle marks just-suprathreshold response for the distal stimulus when the proximal bias was simultaneously just-subthreshold for its own spike. ***E,*** Same cell as (***D***), with proximal and distal roles reversed. Square and pentagon correspond to just sub- and just supra-threshold response peaks, respectively, also shown in (***D***). ***F, G,*** Combined results of 294 stimulus pairs in 6 cells (cell-by-cell results shown in Figure S2 in Text S1). Inset, stimulus sites are indicated by black triangles (electrical stimulation) and purple clouds (laser uncaging), dendrite length in ball-and-stick cartoon is 275 µm. Blue case is same as in (***A*** *–* ***E***). Red case included TTX (1 µM) perfused from an electrode near the soma to prevent somatic spiking which would have masked the subthreshold integration process being studied. Grey and orange cases used electrical stimulation at proximal site instead of uncaging, and included CNQX (10 µM) in the bath to block AMPAR responses in order to prevent somatic spiking due to fast AMPA currents.

Using methods analogous to those in Figures 3 and 4, we carried out two-input summation experiments in brain slices. Proximal and distal sites were activated separately and together over a range of stimulus intensities until an NMDA spike was generated at each stimulus site. In some experiments (i.e., when needed), CNQX was applied in the bath or TTX was applied at the soma to prevent somatic spiking, which would have otherwise occluded the underlying synaptic summation effects. Example traces are shown in Figure 5A for increasing stimulus intensity at the distal electrode, including a distally-evoked dendritic spike. A similar progression is shown for the proximal site in Figure 5B, as well as for the same progression of distal inputs in the presence of a constant proximal stimulus (Figure 5C; the proximal input level in this case corresponded to the asterisked curve in Figure 5B).Summary plots for this cell are shown in Figure 5D and E where the peak somatic depolarization is plotted as a function of the distal or proximal driver stimulus intensity, respectively. The curve families in Figure 5D and E are analogous to slices through the 3-D plots of Figure 4, where different input-output curves correspond to increasing levels of modulation at the second site (as in Figure 1).Note that the assignment of driver and modulator labels to the two sites was arbitrary, and simply reflected the direction in which the data was sliced and plotted. The set of modulation levels used in Figure 5D corresponds to the lowest (dashed) curve in 5E, and vice versa. Triangles indicate the point where a distal spike alone was generated (with zero proximal input), while the transition from square to pentagon indicates the proximal spike alone (with zero distal input). The circle marks the just-suprathreshold response for the distal stimulus when the proximal bias was just-subthreshold for its own spike.

**Figure 6:**
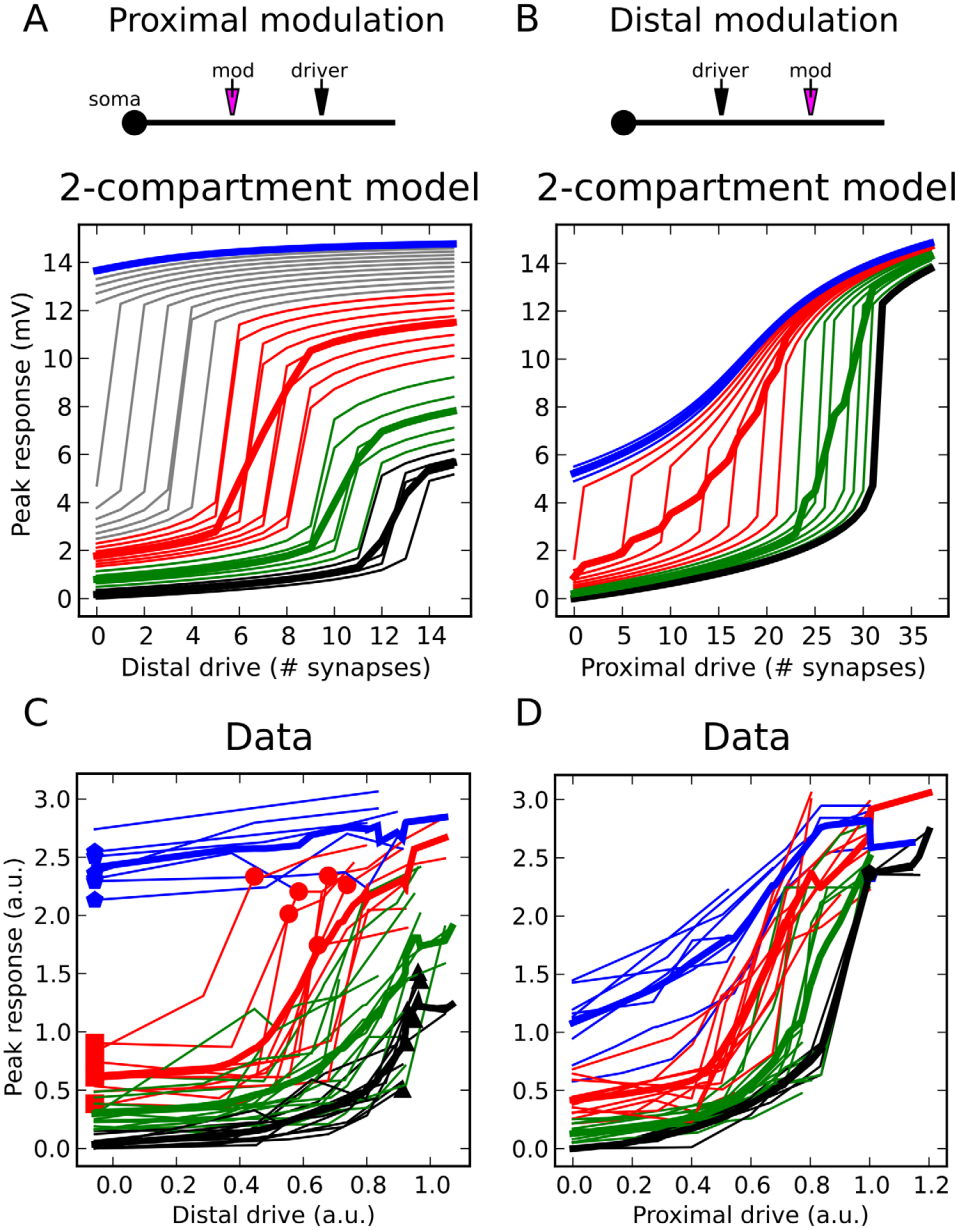
Experimental results match model predictions. ***A, B,*** Orthogonal views of 3D surface shown in Figure 4D for the 2-compartment model. Curves are grouped into 4 categories (colors) based on modulation strength. Averages within each category are shown in bold. Gray curves in (***A***) were excluded from averaging, since corresponding experimental cases were not observed. ***C, D,*** Data from Figure 5F,G was scaled vertically and horizontally using fiducial points for each cell (triangle, square, pentagon, circle; see Materials and Methods) to allow comparison to model results despite different stimulus locations, efficacy, branch input resistances, etc.

Figure 5F and G .show 294 2-input summation cases from 6 cells (the same data is broken down by cell in Figure S2 in Text S1).The substantial variation seen in local spike thresholds and response magnitudes, as indicated by the scatter of like symbols, could be attributed to differences in the locations of the stimulating electrodes, differences in the relative efficiency of electrical stimulation vs. laser uncaging, and substantial cell-to-cell variation in branch diameters. To facilitate comparison of the data to model predictions, which were generated with fixed electrode locations chosen to match the average NMDA-spike responses at proximal and distal locations in our experiments, we normalized the data using fiducial points for each cell corresponding to the four symbols in Figure 5D, E(see Materials and Methods). Data with and without pharmacological blockers was combined given that a case-by-case analysis revealed no systematic difference in the shapes of the input-output curves under the different conditions. Individual curves (thin lines) in the normalized data were color-coded to indicate coarse levels of modulation intensity, from low (black) to high (blue) (Figure 6). Average curves within in each color-coded set are shown as bold lines to facilitate comparison between model and data.

The model and experimental data sets were very similar in form (compare top and bottom rows of Figure 6). Confirming the pattern evident in Figure 5D, the proximal input, when viewed as the modulator, both lowered the threshold and increased the magnitude of the distally-driven input-output curve (Figure 6A, C, see progression from black to green to red curves). Slicing the same data in the orthogonal direction as was done in Figure 5E, the distal input when viewed as the modulator initially lowered the threshold of the proximally driven response (i.e. left-shifted the input-output curve) without increasing its magnitude, eventually leading to a flattening/linearization of the proximal input-output curve at high levels of modulation.

### Proximal-distal summation in the spike-rate regime: model predictions

**Figure 7:**
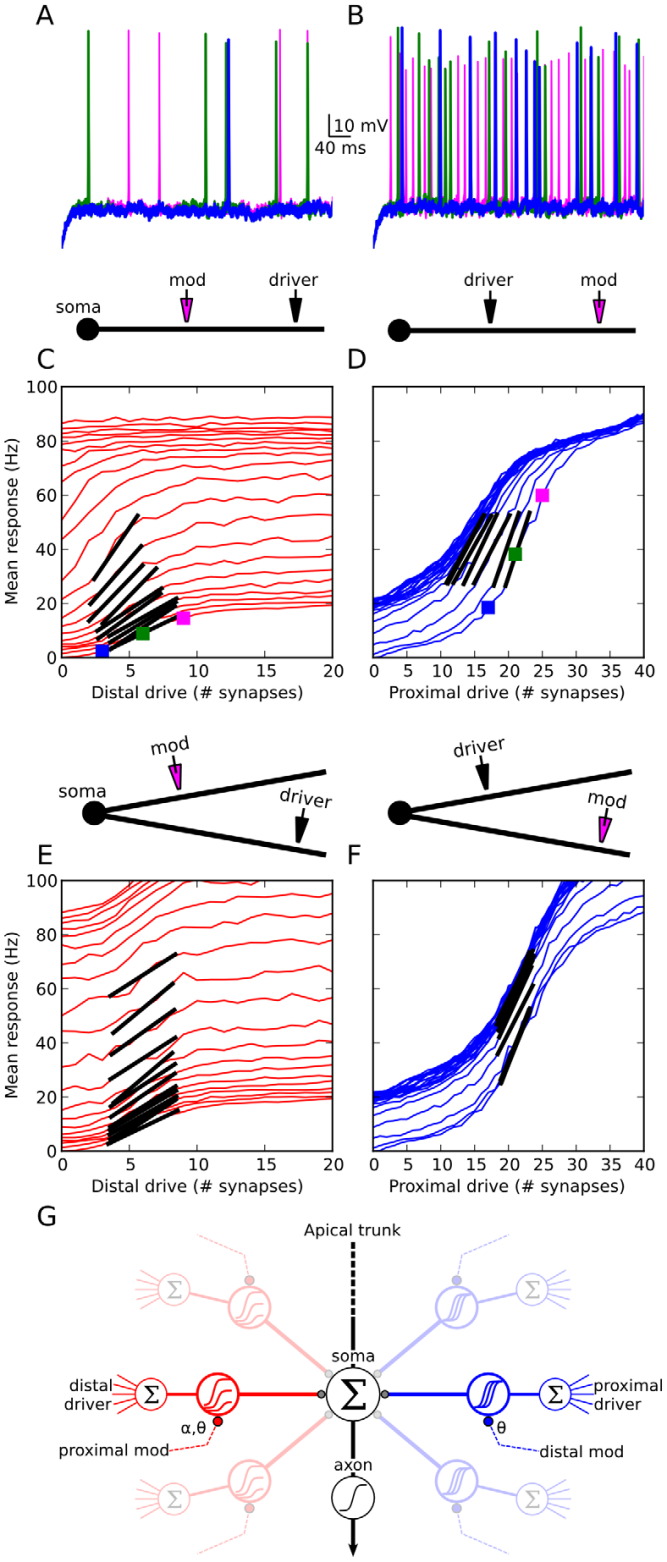
Model predictions of spike rate responses also show strong proximal-distal asymmetry. ***A,B,*** Somatic responses to 50 Hz independent Poisson inputs delivered to 3 (blue), 6 (green), and 9 (magenta) distal synapses centered at 190 µm in (***A***) and 17 (blue), 21 (green), and 25 (magenta) proximal synapses centered at 90 µm in (***B***). ***C*** *,* Mean firing rates for distal drive with proximal modulation increasing from curve to curve (averages of 20 runs). Slope changes are accentuated by black bars centered at point of maximum slope. Colored squares correspond to traces in (***A*** *–* ***B***). ***D,*** Same as (***C***), but for proximal drive with distal modulation. Black bars accentuate left shifting of i-o curve. ***E, F,*** Similar input configuration to (***C*** *–* ***D***), but with proximal and distal inputs (same distances) on two different dendrites. Modulatory effect from both perspectives is linear, as evidenced by the nearly constant additive (vertical shifting) effect of either proximal or distal cross-branch modulation acting on the driver's input-output curves. ***G,*** Diagram illustrates driver-modulator interaction shown in (***C***). Proximal synapses when viewed as contextual modulators (left) lower the threshold θ and increase the gain α of the dendritic sigmoid nonlinearity. Distal synapses viewed as modulators (right) exert a left-shifting (threshold lowering) effect. Note diagrams are schematic representations of the modeling results; absolute and relative positions of the driver and modulator inputs in the schematics should not be given a literal spatial interpretation.

The close match between experimental data and modeling results in Figure 6 indicates that the models capture key features of proximal-distal summation under conditions where the neuron remains subthreshold for somatic spike generation. We ran additional simulations to examine the cell's input-output behavior under more realistic *in vivo*-like conditions where the neuron was driven to fire action potentials. 50 Hz independent Poisson spike trains were delivered to two groups of synapses at 90 and 190 µm from the soma (similar to the location pairs in Figures 4C and 6A,B), and output spike rates were recorded at the soma over a 500 ms period. Spike timing was asynchronous both within and between synapse groups. To enable a single dendrite to drive output spikes, the soma was biased with a noisy current injection that produced a ∼1 Hz background firing rate (see Materials and Methods).Somatic responses are shown for separate activation of the distal (Figure 7A) and proximal (Figure 7B) sites at 3 stimulus intensities. Lower plots show the effect of increasing proximal modulation on distal input-output curves (Figure 7C) and vice versa (Figure 7D). Colored squares correspond to traces in Figure 7A, B.

Despite the much more complex dynamics in these spiking simulations compared to the subthreshold case, the results were overall very similar. The proximal input when viewed as the modulator both lowered the threshold and increased the gain of the distally-driven input-output curve (see black bars in Figure 7C), whereas a distal input when viewed as the modulator only lowered the threshold but did not increase the gain of the proximal input's input-output curve (Figure 7D).

To verify that the nonlinear proximal-distal interaction shown in Figure 7C, D reflected a *bona fide* within-dendrite effect, we ran control simulations in which proximal and distal inputs were delivered to two different dendrites. In contrast to the nonlinear within-branch interactions, firing rates generated by the separate branches combined nearly linearly at the soma (Figure 7E, F), consistent with linear between-branch summation reported in previous studies [28], [30]. We also found that nonlinear within-branch interactions remained very similar when proximal and distal inputs were distributed “regionally” across multiple branches of a dendritic subtree rather than limited to a single branch (Figure S3 in Text S1). Overall, the pattern of asymmetric summation between proximal and distal sites found under simulated *in vivo*-like conditions closely paralleled the subthreshold results.

### Evidence for proximal-distal segregation of input pathways on pyramidal neuron dendrites

**Figure 8:**
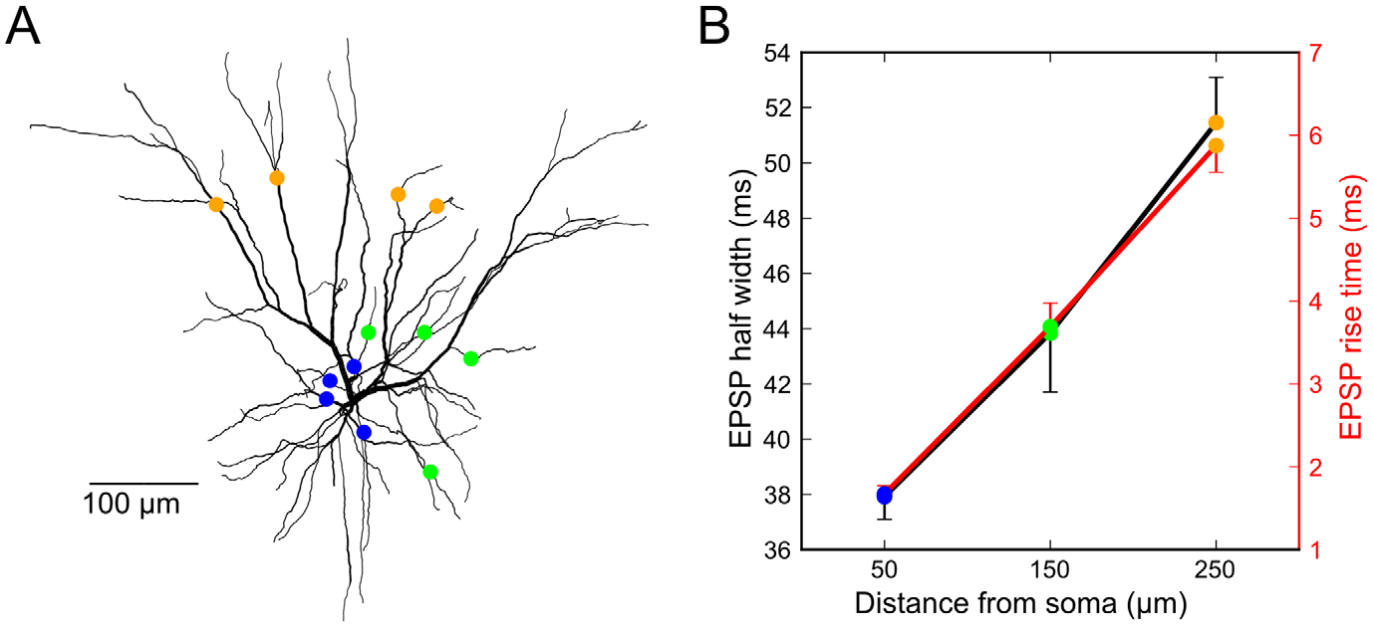
EPSP time-course analysis of L3 model neuron suggests that “modulatory” long-distance horizontal connections terminate proximally and vertical L4 “driver” inputs terminate distally. ***A,*** L3 model morphology [89, see Materials and Methods for details] with colored markers indicating one set of the locations of the 4 synapses evoking the responses shown in (***B***). ***B,*** Four synaptic inputs were placed on 100 sets of 4 randomly selected dendrites 50, 150, and 250 µm from the soma, evoking 4.6, 3.6, and 2.9 mV EPSPs on average. EPSP half width and risetime grew with distance from the soma. Error bars indicate s.d. of the mean across random dendritic sets. Compare to Table 1 from Yoshimura et al. [47] showing that EPSP half widths between modulatory� long-distance horizontal and vertical L4 “driver” inputs increased (34.5±19.9 vs. 53.0±28.1ms, p<0.05, t-test) as did EPSP rise times (3.9±2.5ms vs. 5±2.5ms, p<0.04, Wilcoxon rank-sum test, Figure 2B rise time data from Yoshimura et al. [47] was digitized with Engauge Digitizer for statistical analysis in Matlab).

Pending the availability of anatomical “connectome” data that establishes whether pathway-specific biases exist in the spatial targeting of excitatory synapses onto PN basal dendrites, a pathway's tendency to terminate proximally vs. distally can potentially be distinguished by electrophysiological measures. In particular, cable theory predicts that somatic EPSPs generated by proximal synapses will have faster rise times and narrower half widths than similar synapses activated distally [37], [46]. In a possible example of this effect, data of Yoshimura et al. [47] from kitten visual cortex suggests that vertical inputs from layer 4 onto layer 2–3 pyramidal neurons, and long-range horizontal (LH) connections between layer 2–3 PNs, may terminate at different distances from the soma. Unitary EPSPs evoked by LH axons connecting layer 2–3 pyramidal cells at separations of 350 to 1000 um had significantly shorter half widths (34.5±19.9 vs. 53.0±28.1 ms, p<0.05, t-test) and faster rise times (3.9±2.5 ms vs. 5.0±2.5 ms, p<0.04, Wilcoxon rank-sum test) than unitary EPSPs evoked by stimulation of vertical inputs from layer 4. Consistent with the predictions of cable theory, in compartmental simulations designed to replicate the Yoshimura et al. data (see Materials and Methods), we found that the shorter half widths and faster rise times for LH connections, compared to longer half-widths and slower rise-times for vertical inputs from layer 4,suggests that as a population, the LH connections in primary visual cortex target more proximal dendritic locations than do vertical inputs (Figure 8). In light of our findings here (Figure 7), these LH connections, which are generally thought to carry contextual information, would be expected to exert a gain-boosting effect on cell responses driven by the vertical inputs from layer 4. This is consistent with reports of multiplicative boosting of classical receptive field responses in visual cortex by horizontally offset contextual cues [4], [48].

## Discussion

Using a combined modeling and experimental approach we have identified a new biophysical mechanism tied to synapse location on basal dendrites that could provide a basis for asymmetrical interactions between classical and contextual inputs to neocortical pyramidal neurons. The mechanism depends on the increase in input resistance and voltage attenuation for stimulus sites at increasing distances from the soma, and on the voltage-dependence of NMDA channels, but does not depend critically on synaptic time courses, cable delays, or other aspects of membrane dynamics. A variety of differences other than synapse location may also contribute to nonlinear classical-contextual interactions in the neocortex, including different neurotransmitter receptor subtypes [49], [50], different short-term synaptic dynamics [16], [51], or different presynaptic firing patterns [52]. In the location-dependent mechanism we have identified here however, even if synaptic properties and activation patterns are everywhere the same, the differential biasing of synaptic projections along the proximal-distal axis of PN basal dendrites provides a simple and flexible means for excitatory inputs to PNs to exert a spectrum of classical-contextual interactions (Figure 7). Modulatory pathways designed to lower the threshold of classical RF responses would either co-terminate with, or target more distal sites than their associated driver inputs (Figure S4A in Text S1). Modulatory pathways designed to boost response gain, including attentional inputs [7], [53], contextual cues in the extraclassical receptive field [4], [48], [54], [55], and extrasensory inputs underlying gain field effects [9], [10], would, according to this view, preferentially target more proximal sites than their corresponding classical driver inputs (Figure S4B in Text S1).

### Relations to previous work

Previous studies in layer 5 and CA1 pyramidal neurons have mostly focused on nonlinear interactions between proximal and distal inputs to the apical dendritic tree [18]–[20], [22], [41]. Though these studies like ours are concerned with nonlinear synaptic interactions in PN dendrites, our findings and conclusions can be distinguished from earlier work in two major respects. First, the biophysical mechanisms we describe differ from previously reported mechanisms. Oakley et al. [41] explored the interaction of glutamate-evoked calcium plateau potentials (>500 ms) evoked at different points along the apical trunk of a layer 5 pyramidal cell; the main proximal-distal effect reported was that the response to a combined proximal and distal input was dominated by the proximal response, whereas the distally-evoked response was largely occluded. Other studies have focused on the coupling between sodium and/or calcium spike-generating mechanisms in the distal apical tuft region and the cell's main firing mechanism at the soma [18]–[20], [22]. The Larkum et al. [19] and Jarsky et al. [22] studies focus on the gating of distally-generated sodium spikes travelling to the soma, where the gating was controlled by a depolarizing input that “rescues” the forward-propagating spike at a point on the path to the soma where it would otherwise fail. Other work [18], [20] has examined the modulatory role played by dendritic calcium spikes. When a somatic action potential back-propagates into the apical tree and combines with a distal depolarizing current injection to trigger a dendritic calcium (BAC) spike, the resulting current then flows “back” to the soma to produce a burst of somatic action potentials. The number of spikes in that burst, vs. the single spike that triggered it, can be thought of as the multiplier that accounts for the cell's overall gain increase [20]. Remondes and Schuman [21] and Takashashi and Magee [23] showed similar coupling effects involving mixed fast and slow spikes evoked by temporoammonic input to the distal apical trees of CA1 pyramidal cells, where the coupling of these distal regenerative events to somatic firing was enabled by NMDA currents activated more proximally in the stratum radiatum [see also 56].Even more complex apical-somatic coupling effects involving timing and inhibitory circuits have also been reported [57], [58].

In contrast to these previously described mechanisms involving inputs to the main apical trunk, and/or coupling of distal Na^+^ and Ca^2+^ spikes to the soma through the main apical trunk, the location-dependent summation nonlinearity we report here depends on (1) the voltage-dependence of synaptically activated NMDA channels, previously shown to be the major regenerative current carriers in PN thin dendrites [32], [39], [40], [44], [52], and (2) the asymmetric cable properties that result when a thin dendrite connects to a larger trunk or soma [36]. Interestingly, Branco et al. [33] have recently shown that a PN's ability to distinguish excitatory stimulus sweeps towards or away from the cell body on a basal dendrite also depends on an interaction between NMDA currents and spatially-varying cable properties. This suggests that similar biophysical building blocks may contribute to very different forms of nonlinear synaptic integration.

The second major respect in which the location-dependent summation mechanism we have described differs from previously reported integrative mechanisms in PNs is the level of spatial resolution. Synaptic interactions mediated through the main apical trunk, especially when they involve coupling of distal apical and somatic spiking mechanisms [18]–[23] are acting on a relatively global scale within the dendritic arbor. In contrast, the mechanism we have identified operates within the confines of individual thindendrites [see also 33] – and could potentially account for modulatory effects that operate on a finer than receptive field scale [4], [59], [60]. This biophysical capability, if exploited by PNs, could explain how attentional [59], [60] and contextual [4] influences can selectively alter the responsiveness of a single receptive field subunit within a multi-subunit “complex” cell in the cortex [4], [59], [60]. In order for neurons to take advantage of subunit-specific modulation effects, driver inputs representing different stimulus variants – different receptive field positions, different color channels, etc. – would need to be segregated onto different dendritic branches [26], [27], [61] so that they could be separately targeted by modulatory pathways. A recent report that used *in vivo* optical recording of Ca^2+^ signals to study inputs to the dendrites of orientation-tuned neurons in mouse visual cortex came close to addressing this issue [62], but did not reach the question as to whether the different dendrites of an orientation-tuned neuron differ in some feature other than orientation, such as different receptive field locations. Some evidence has been found for spatial segregation within the dendritic trees of sensory neurons [63], though direct evidence that such segregation occurs in the neocortex, and on what spatial scale, is currently lacking.

### Driver-modulator segregation on pyramidal neuron basal dendrites

Though our study has focused on 2-input summation effects within a single dendrite, this does not imply that each dendrite necessarily processes unique classical and/or contextual signals: the same classical and contextual pathways might project to multiple basal dendrites or the tree as a whole, while maintaining their segregation in the radial dimension. Direct evidence for excitatory pathway segregation even at this coarser level of resolution is also lacking, but has a strong precedent: the targeting of different synaptic pathways to different dendritic zones is the rule rather than the exception in CNS organization [35], [64], [65], a rule that certainly applies to pyramidal neurons in other respects: apical tuft dendrites are innervated by different axons than basal dendrites both in the neocortex [66]–[70] and hippocampus [71], and within the basal arbor itself different classes of interneurons are known to selectively target somatic-perisomatic vs. distal sites [72]–[74], just as we propose here for excitation. Furthermore, a spatial segregation of classical and contextual excitatory inputs to basal dendrites would likely depend on location-dependent neural plasticity mechanisms. In keeping with this, Gordon et al. [75] recently showed that the rules for synaptic long-term potentiation are different at proximal vs. distal sites on pyramidal neuron basal dendrites [see also 76], [77]. Confirmation or refutation of the modulation-by-location hypothesis will require high-resolution anatomical and physiological mapping techniques capable of identifying the major sources of excitatory synapses onto PN basal dendrites, including long-range horizontal and cortico-cortical connections [65], [78]–[81], in conjunction with physiological recordings of somatic and dendritic potentials under varying states of response modulation *in vivo* [82], [83]. To the extent that excitatory projection biases onto PN basal dendrites are found, the present framework will be of help in interpreting the functional consequences of such biases for cortical circuit computations.

### Implications for dendrites with normalized synapses

The nonuniform cable properties of thin dendrites connected to main trunks or the soma mean that synapses at more proximal sites experience lower input resistances than their distal counterparts, so that a larger number of (equivalent) synapses is needed to push the membrane at a proximal site into the NMDA voltage-dependent regenerative range compared to a distal site (Figure 4B,D). It is thus interesting to note that on apical oblique dendrites of CA1 pyramidal cells, recent evidence indicates that spine volumes and PSD areas are largest near the proximal ends of the branches and grow systematically smaller moving distally, suggesting that excitatory synaptic conductances are at least partially normalized to the local input resistance [84]. Such a scheme would help equalize the stimulus intensity requirements for pathways projecting selectively to proximal vs. distal sites on these branches, though it is not currently known whether this form of pathway segregation occurs in CA1.

### Similarity of excitatory and inhibitory location effects

The location-dependent excitatory effects reported here are intriguingly similar in form, though opposite in direction, to location-dependent inhibitory modulation effects we have recently described in these same dendrites [85]. In that related study, we found that inhibitory inputs to PN basal dendrites also differently affect a dendrite's sigmoidal input-output curve depending on their location: a distal inhibitory input increases the threshold for an NMDA spike triggered by a more proximal input, that is, it right-shifts the proximal input's sigmoidal response curve. In contrast, a proximal inhibitory input both increases the threshold and lowers the gain of the sigmoidal response to a more distal input, analogous, but opposite, to the combined threshold and gain effects associated with proximal excitatory modulation. The very similar form of these excitatory and inhibitory modulation effects strengthens the case that PN thin dendrites, by virtue of their voltage-dependent NMDA currents and asymmetric cable properties, possess significant nonlinear analog processing capabilities tied to synapse location [33], [39], [44]. These include the ability for excitatory and inhibitory modulatory pathways to bi-directionally manipulate the thresholds and gains of dendritic input-output curves through biases in the spatial distribution of their synaptic influences along the proximal-distal axis of perisomatic thin dendrites. In the case of excitation, biases would be set up in the direct excitatory projections onto PN dendrites. In the case of inhibition, biases would be established indirectly by manipulating a pathway's relative activation of dendrite vs. soma-targeting interneurons.

### “Dark computation” in the neocortex?

If in future experiments systematic variations in excitatory synapse distributions on PN thin dendrites are determined to play a significant role in mediating classical-contextual interactions, it is understandable how such a location-based computing mechanism could have escaped notice up to this point. Unlike other modulation mechanisms in which driver and modulator synapses are distinguishable based on measurable physical characteristics, such as synapse size or post-synaptic receptor type [35], modulation-by-location in its pure form would be locally invisible (i.e. “dark”), in the sense that under the microscope, dendrites would appear to be lined with an undifferentiated population of excitatory synapses. Only when the remote source of each synaptic contact has been traced, could the nature – or even the existence – of the location-based computation be inferred. The possibility that analog location-dependent computations do routinely occur within the dendrites of neocortical PNs, that contribute to the modulation of PN response by a multitude of attentional, contextual, and cross-modal influences, highlights the continuing need for multi-disciplinary approaches in analyzing neocortical circuits.

## Materials and Methods

### Multi-compartment modeling

All simulations were performed using the NEURON modeling package (version 7.0 r276) [86]. Unless otherwise indicated, all simulation studies utilized a 3D reconstructed layer 5 pyramidal cell morphology (see Figures 2E inset and 4A) that was a smoothed version of the “j4” morphology [87], [88], to which a myelinated axon was added to model axonal spike initiation [89]. Ion channel models and distributions were constrained by a variety of data [28], [30], [38], [90], [91]. Parameters are shown in Table 1. NEURON files are available upon request.

**Table 1:** Model parameters.

	Property	Value	References
**Passive Properties**	R_m_	dendrites: 10 kΩ cm^2^	[89]
****		axon nodes: 50 Ω cm^2^	[89]
		other: 20 kΩ cm^2^	[89]
	C_m_	dendrites: 2 µF/cm^2^	[89]
		myelinated axon: 0.05 µF/cm^2^	[89]
		other: 1 µF/cm^2^	[89]
	R_a_	100 Ωcm	[89]
	E_leak_	−70 mV	
**Active Properties**	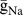	dendrites: 0.006 S/cm^2^	[38]
****		non-myelinated axon: 5 S/cm^2^	[38]
		myelinated axon: 0.006 S/cm^2^	[38]
		soma: 0.25 S/cm^2^	[38]
	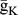	dendrites: 0.0003 S/cm^2^	[29]
		non-myelinated axon: 0.05 S/cm^2^	[29]
		soma: 0.03 S/cm^2^	[29]
	E_Na_	+60 mV	
	E_K_	−90 mV	
**Synapses**	AMPAR	g_max_ = 1.5 nS	[38], [90], [91]
		τ_rise,fall_ = 0.05, 0.5 ms	[38], [90], [91]
	NMDAR	g_max_ = 3.56, or 3.9 nS	[30], [92], [93], [95]
		τ_rise,fall_ = 2.1, 18.8 ms	[30], [92], [93], [95]
	E_AMPA/NMDAR_	0 mV	

Excitation was delivered at varying distances from the soma through combined NMDAR/AMPAR type synapses. The AMPA component in each synapse had a fixed peak conductance while the NMDA peak conductance doubled (2.23 to 4.46 nS) from the first to second pulse in 50 Hz double-pulse stimulation experiments (Figures 3–6).Values were fit based on measured physiological summation nonlinearities for single and double pulse stimulation experiments [30], were in keeping with increases in NMDA conductance upon repeated stimulation [92], and non-saturation of the NMDA receptor [93]. Both AMPA and NMDA conductances were modeled as difference-of-exponential functions with kinetics appropriate for 35°C (see Table 1). The NMDA channel model included an instantaneous voltage-dependent Mg-block of the form B(V) = 1/(1+e^−(V+12)/10^). Hodgkin-Huxley style sodium and potassium conductances were included in the axon, soma and dendrites, with the sodium conductance decreasing linearly to zero at a distance of 200 µm from the soma [38]. For single-pulse simulations mimicking single pulse UV glutamate uncaging in Figure 2, NMDA peak conductance was set to 3.56 nS to match the NRLE of the *in vitro* data.

Synapse clusters were centered at specified locations with 0.5 µm spacing [34]. Terminal dendrites were corrected for the membrane area contribution of unmodeled spines by increasing membrane capacitance and conductance by a factor of 2.0 [34]. In simulations with NMDAR block, the NMDA channel peak conductance was set to 0.

The axon, soma, and all dendritic subtrees containing activated synapses were divided into electrical compartments, or “segments” of length no greater than one tenth of the section's length constant at 100 Hz [94], or 10 µm - whichever was smaller. In other dendrites, 3 segments were used per section without loss of simulation accuracy.

Suprathreshold (spike rate) results used the same model as above except for the excitation which was in the form of unsynchronized 50 Hz Poisson trains, and the peak NMDA conductance was fixed to 3.9 nS. To achieve a low background firing rate (∼1 Hz), the axo-somatic spike generating mechanism the soma was biased with a noisy current injection (0.75±1 nA) updated every integration time step (0.1 ms).Spike rates were averaged over the 500 ms stimulus period.

### NRLE measure

For each data point (x_i_, y_i_) beginning with the second point on each input-output curve, a line was fit to all preceding data points, and extrapolated to the point (x_i+1_, y_extrap_). The ratio of the actual y value to the linearly extrapolated y value y_i+1_/y_extrap_ was computed, and the maximum of this ratio along a given input-output curve was taken as the NRLE for that curve.

### EPSP time-course analysis

The EPSP study in Figure 8 utilized a published L3 model [89] with the following changes: 1) Spine correction was changed to be the same as described above, which does not distort the morphology, enabling our analysis of EPSP properties versus distance, and 2) R_m_ and C_m_ were increased by a factor of 1.6 so that the EPSP half-width ranges in the model were similar to those in Yoshimura et al. [47]. Synapses were AMPA-type only and were modeled as difference-of-exponential functions with τ_rise,fall_ = 0.2,2 ms and 2 nS peak conductance. EPSP properties were similar when synapses contained mixed NMDA/AMPA conductances similar to those used elsewhere in the paper (data not shown).

### 2-compartment circuit analysis

Time-invariant voltage responses were calculated using methods described elsewhere [85], but with the addition of a second NMDA conductance (see Figure 4C). The Kirchhoff's current law equations were as follows:
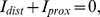
where
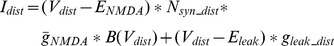
and
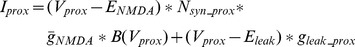
(1)By exploiting the relationship between V_prox_ and V_dist_:
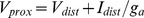
(2)to eliminate the dependence on V_prox_ in Eq. (1) the resulting equation was solved numerically for V_dist_, then V_prox_ was computed using Eq. (2). Here, B(V) = 1/(1+e^−(V+22)/12^), slightly ‘softer’ than the magnesium block term used in the multi-compartment model. This was done to account for the “dilution” of the NMDA voltage-dependent non-linearity by co-activated AMPA channels, which were not explicitly included in the 2-compartment model. In Figure S1 in Text S1, CTO, the ‘current-to-overcome’, was defined as –(I_soma_-I_NMDA_Drive_), corresponding to the ‘net leak’ in the control and modulation conditions.

### Slice preparation and electrophysiological recording

Neocortical brain slices 300–350 µm thick were prepared from 18- to 28-day-old Wistar rats. All experimental procedures were in accordance with guidelines of the Technion Institutional Animal Care and Use Committee. Extracellular solution contained 125 mM NaCl, 25 mM NaHCO3, 25 mM glucose, 3 mM KCl, 1.25 mM NaH2PO4, 2 mM CaCl2 and 1 mM MgCl2 (pH 7.4) at 35–36°C. Intracellular solution contained 115 mM K^+^-gluconate, 20 mM KCl, 2 mM Mg-ATP, 2 mM Na2-ATP, 10 mM Na2-phosphocreatine, 0.3 mM GTP, 10 mM HEPES and 0.15 mM Calcium Green-1 (CG-1) or 0.2 mM Oregon Green 488 Bapta-1 (OGB-1), pH 7.2. GABA_A_ receptor blocker bicuculline methiodide (BCC; 1–20 µM) was added to the extracellular solution in some experiments. Whole-cell patch-clamp recordings were made from visually identified layer-5 pyramidal neurons using infrared-differential interference contrast optics. Electrophysiological recordings were performed using Multi-Clamp 700A (Axon Instruments, Foster City, CA), and the data were acquired and analyzed using Pclamp 8.2 (Axon Instruments), Igor (Wavemetrics, Lake Oswego, OR), and in-house software. All statistical analyses used the Student's t-test.

### Focal stimulation

The neurons were filled with a calcium-sensitive dye (CG-1 or OGB-1) and the basal dendritic tree was imaged with a confocal imaging system (Olympus Fluoview) mounted on an upright BX51WI Olympus microscope (Tokyo, Japan) equipped with a 60× (0.9 n.a.; Olympus) water objective. The theta stimulating electrodes were filled with Alexa Fluor 647. Full images were obtained with a temporal resolution of 1 Hz and in the line scan mode with a temporal resolution of 512 Hz. Images were analyzed using Tiempo (Olympus), Igor (Wavemetrics), and in-house software. Focal synaptic stimulation was performed with a theta patch pipette located in close proximity to the selected basal dendritic segment, as guided by the fluorescent image of the dendrite. We limited ourselves to dendritic regions that were more distal than the initial 50-µm segment of the basal dendrites, as we could not obtain focal synaptic activation in those regions.

### Glutamate uncaging

For the uncaging experiments, caged glutamate (4-methoxy-7-nitroindolinyl(MNI)-glutamate; Tocris, San Diego, CA) was photolyzed with a 361 nm UV-laser beam (Enterprise 2; Coherent, Palo Alto, CA) using point scan mode. The caged glutamate (5–10 mM) was delivered locally to a branch using pressure ejection (5–10 mbar) from an MNI-glutamate-containing electrode (2 µm diameter).

### Normalization of two-input data using a fiducial point template

The four fiducial points indicated by shapes in Figure 5D,E were used to normalize the data from each of the 6 cells (Figure 5F,G). The normalization results are shown in Figure 6 C,D. The square and pentagon indicate just-subthreshold and just-suprathreshold responses for the proximal spike alone, the triangle indicates the just-suprathreshold response for the distal spike alone, and the circle was just-suprathreshold for the distal stimulus when the proximal bias was simultaneously just-subthreshold for its own spike. We noted that over the data set: (1) the proximal spike was more than twice the height of the distal spike (compare height of pentagon and triangle); (2) the proximal just-subthreshold response was about 2/3 the height of the distal spike response (compare y-coordinates of square and triangle); and (3) the threshold for spike generation by a distal input was roughly cut in half when boosted by a just-subthreshold proximal bias (compare x-coordinates of circle and triangle. Given these observations, we created a template set of fiducial points based on the average ratios found in the experiments: triangle = (1, 1); square = (0, 0.6); pentagon = (0, 2.4); circle = (0.6, 2.2).For any given cell, the 2-D data (distal drive, proximal modulation) was scaled using the horizontal and vertical scaling factors that minimized the MSE between actual and template fiducial points. Note that only 2 scaling factors found through MSE minimization for each cell were used to scale all 29–56 data points for that cell. Overfitting was thus avoided. It is worth noting that we previously attempted a more ‘intuitive’ normalization procedure based on only the two fiducial points corresponding to the proximal and distal spikes, but because that normalization scheme did not capture threshold lowering and spike boosting affects of ‘medium’ strength modulators, that approach resulted in a poorer match between the data and the model. Thus, we used the additional fiducial points which capture more of the relevant features of each experimental data set.

The data viewed from the orthogonal perspective (proximal driver, distal modulation) was more uniform, so that only a single fiducial point was needed for normalization: each plot was rigidly scaled to place the pentagon at the point (1, 2.4) (Figure 6D).

## Supporting Information

Text S1 **Supplementary Figures S1-S4.**
(PDF)
