# Supplementary material for: Location-Dependent Excitatory Synaptic Interactions in Pyramidal Neuron Dendrites
Source: PLoS Comput Biol. 2012 Jul 19;8(7):e1002599. doi: 10.1371/journal.pcbi.1002599 (PMC3400572; doi:10.1371/journal.pcbi.1002599)
Supplement: Text S1 — Supplementary Figures S1-S4. (PDF) [file pcbi.1002599.s001.pdf]

Supplementary Figures

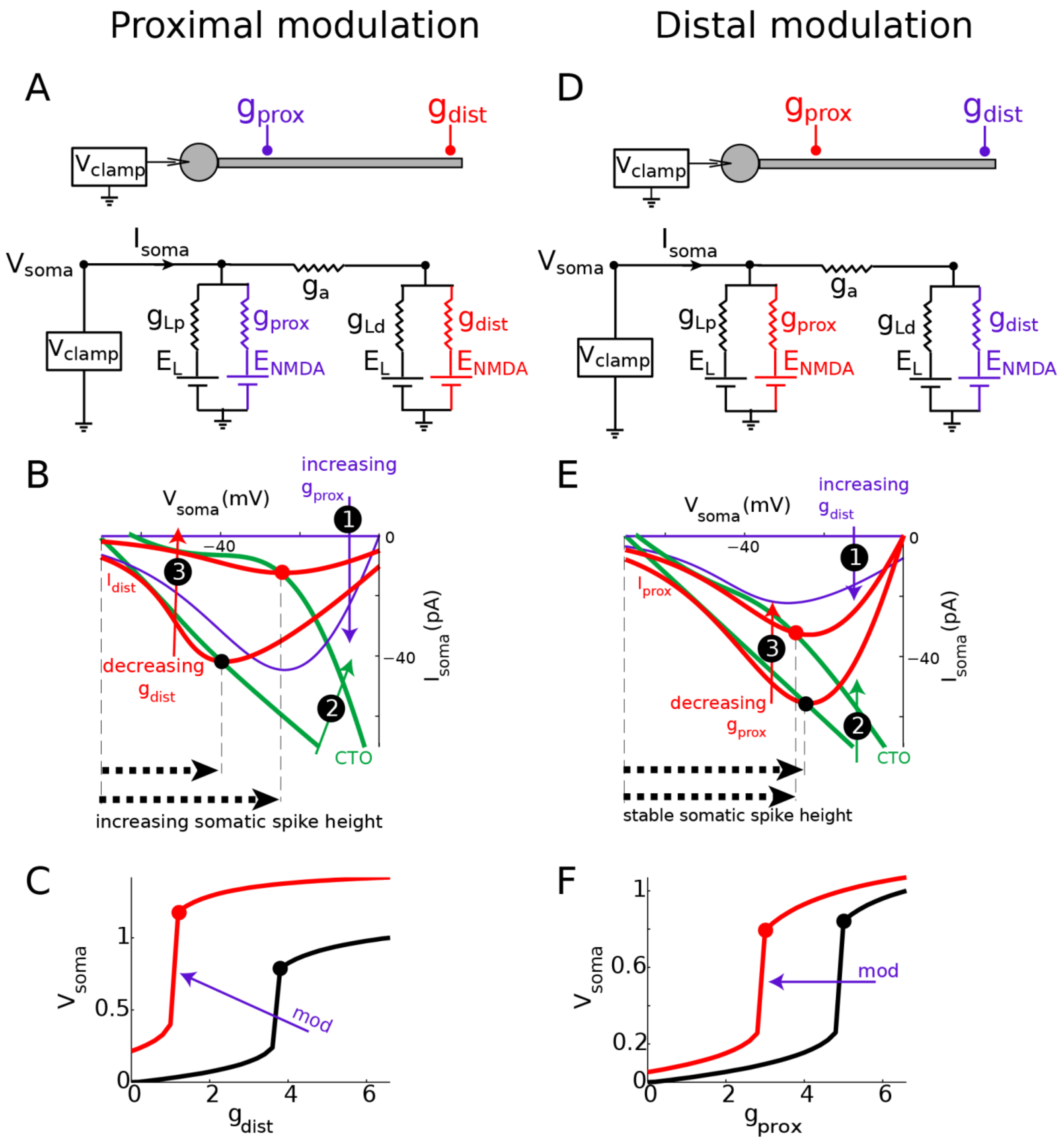

Figure S1. Proximal vs. distal modulation in a 2-compartment model, related to Figure 4C. ***A,*** 2-compartment time-invariant circuit with proximal and distal NMDA conductances. Red indicates the distal site is in the role of driver, purple indicates the proximal site is the modulator (roles are reversed in (***D-F***)). ***B,*** I-V curves measured at soma for the circuit in (***A***). 2 purple curves are I-V curves for g_{prox}: horizontal curve on x-axis is for zero modulation (i.e. “control”) condition, which grows into the standard NMDA I-V curve in the “modulation” condition (see marker #1; the other 2 markers show corresponding transitions in the green and red I-V curves when the modulator is activated).

Green curves labeled CTO represent the “current to overcome” that the driver conductance must produce to generate an NMDA spike.CTO includes all leak conductances and the modulator; the two CTO curves are for control and modulation conditions (marker #2). CTO curves are plotted downward so their magnitudes can be more easily compared to the corresponding driver (red) I-V curves. Two red curves show distal driver I-V curves at spike threshold in both the control and modulation conditions. Spike threshold is reached when the red curve is large enough in magnitude to just skim below the corresponding green CTO curve, so that the stable equilibrium point (the intersection of the red and green curves, marked by a black or red dot) jumps to a value well above rest. The equilibrium point is interpreted as the peak of the NMDA spike, indicated by bold dashed lines. Effect of the proximal modulator is to lower the driver conductance needed to generate a spike (see marker #3), while significantly boosting the spike height. ***C,*** Summary plot shows proximal modulator reduces threshold and increases distally-driven spike height. Black curve is for control, red for modulation condition. ***D,*** Same circuit as (***A***) but with distal input now considered the modulator (purple). ***E,*** Analogous to (***B***), where effect of distal modulator (marker #1) again leads to a lifting of the green CTO curve (marker #2), which again reduces the conductance threshold needed to achieve a spike (marker #3).In this case, the modulation produces a very slight reduction in spike height (dashed arrows). ***F,*** Summary curves analogous to those in (***C***) show reduction in threshold with little change in spike height.

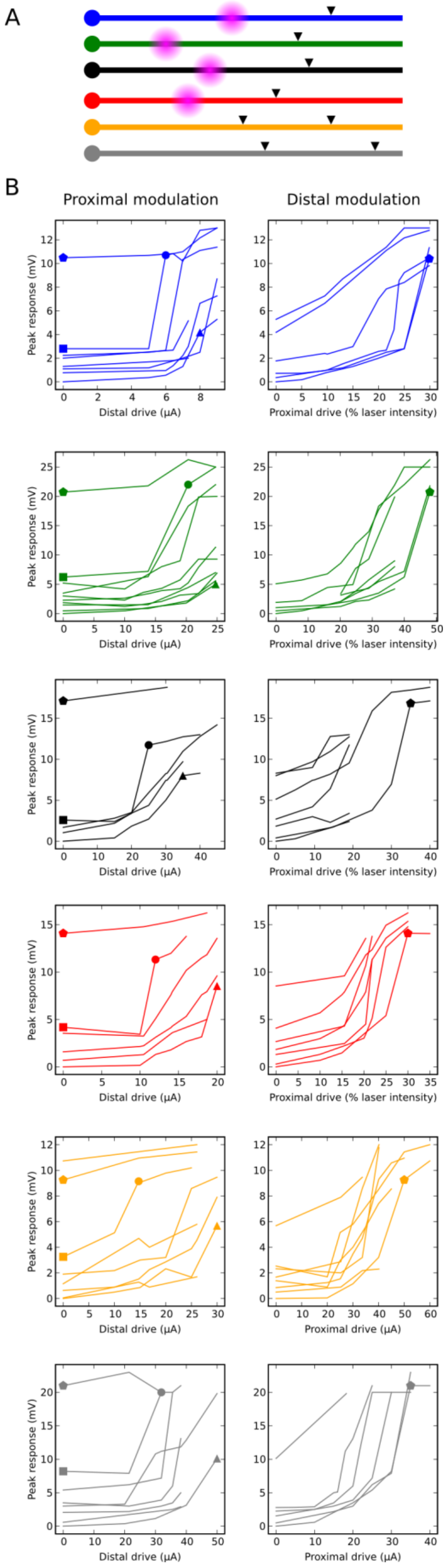

Figure S2. Cell-by-cell proximal-distal interaction data, related to Figure 5. ***A,*** Stimulus pairs in 6 cells; stimulus sites are indicated by black triangles (electrical stimulation) and purple clouds (laser uncaging).Dendrite length in ball-and-stick cartoon is 275 μm. Red case included TTX (1 μM) perfused from an electrode near the soma to prevent somatic spiking which would have masked the subthreshold integration process being studied. Grey and orange cases used electrical stimulation at proximal site instead of uncaging, and included CNQX (10 μM) in the bath to block AMPAR responses, again to prevent somatic spiking – in this case due to fast AMPA currents. ***B,*** Proximal modulation (left) and distal modulation (right) results plotted separately cell-by-cell; same as combined results plotted in Figure 5F,G.

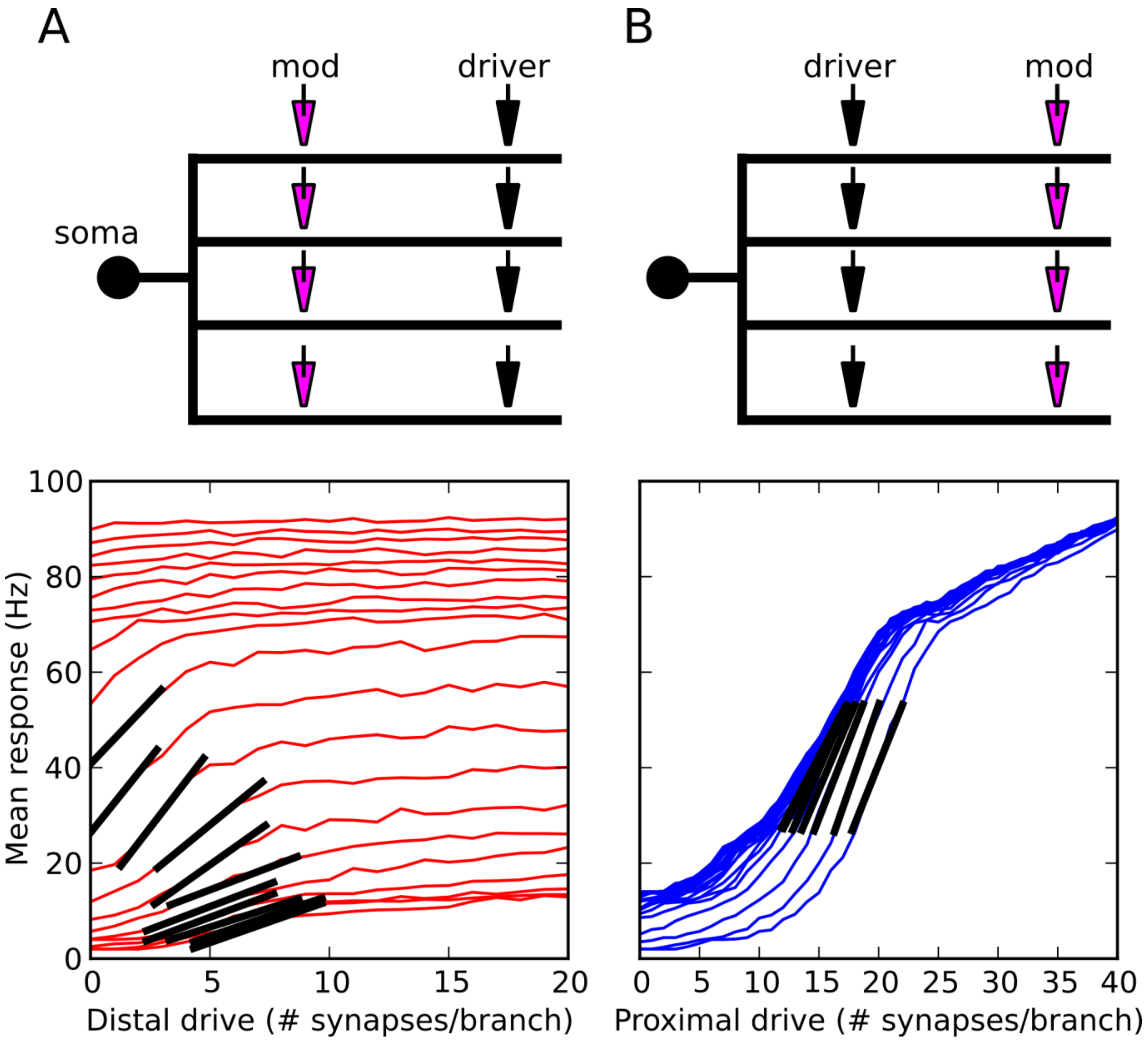

Figure S3. Multi-branch proximal-distal interactions distributed over a dendritic subtree exhibit similar asymmetric nonlinearities, related to Figure 7. ***A,B,*** Same format and stimulus locations as Figure7C,D except that 4 terminal dendrites of a basal subtree were stimulated –as might occur when an afferent pathway targets a dendritic subregion [63]. To maintain the cell's firing rate within the same range, an additional dynamic clamp conductance was delivered to the soma mimicking the divisive effect of background excitatory and inhibitory activity [15].

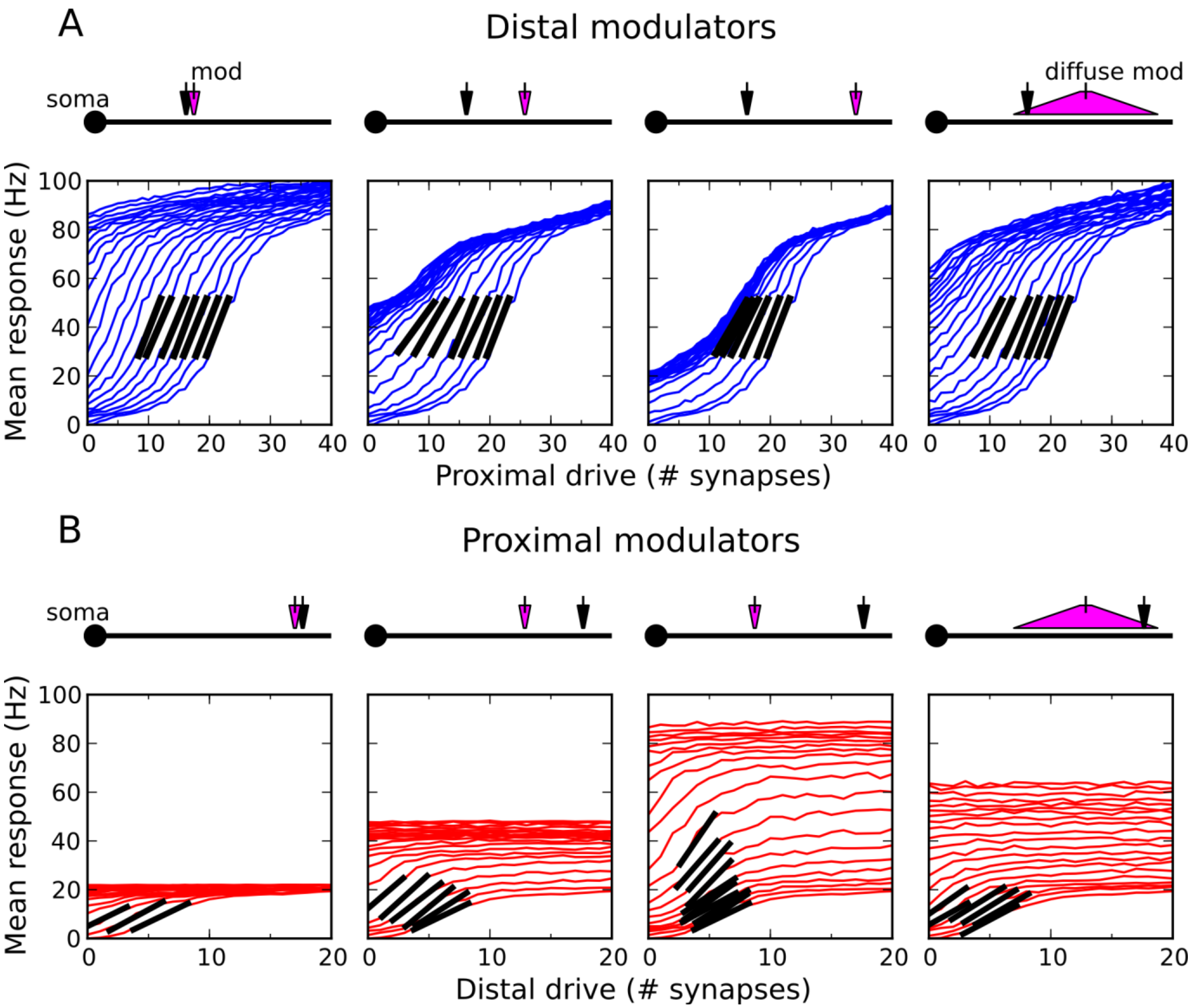

Figure S4. ‘Multiplicative’ modulatory influences should target more proximal sites than co-activated drivers, while threshold-lowering modulation should be either co-localized or more distal than co-activated driver inputs, related to Figure 7. ***A,***(All 4 plots) Distal modulators have similar threshold-lowering effects when co-localized or more distal than co-activated driver inputs. Third column is same as Figure 7C,D.***B,***(First 3 plots) Proximal modulators become progressively more multiplicative as they move more proximally relative to the driver. Focal modulation at the center of the branch (second column) acts similar to diffuse modulation over much of the branch (fourth column).
